# Prevalence and Patterns of Positional Dental Anomalies in First Permanent Molars: Insights from a Study in Oradea, Romania

**DOI:** 10.3390/diagnostics14131460

**Published:** 2024-07-08

**Authors:** Rahela Tabita Moca, Abel Emanuel Moca, Raluca Iulia Juncar, Luminița Ligia Vaida, Anna-Maria Janosy, Mihai Juncar

**Affiliations:** 1 Doctoral School of Biomedical Sciences, University of Oradea, 1 Universității Street, 410087 Oradea, Romania; rahelamoca@gmail.com; 2 Department of Dentistry, Faculty of Medicine and Pharmacy, University of Oradea, 10 Piața 1 Decembrie Street, 410073 Oradea, Romania; ligia_vaida@yahoo.com (L.L.V.); mihaijuncar@gmail.com (M.J.); 3 Private Dental Practice CMI Dr. Janosy Anna-Maria, 23 Corneliu Coposu Street, 410445 Oradea, Romania; ajanosy@yahoo.com

**Keywords:** tooth position anomalies, first permanent molars, Romanian population

## Abstract

Tooth position anomalies, influenced by both genetic and environmental factors, can significantly impact oral health and play a critical role in establishing proper occlusion. The aim of this study was to identify the most prevalent tooth position anomalies in first permanent molars among Romanian patients and to evaluate additional variables as well. This retrospective study utilized digital study models to identify all existing tooth position anomalies. The study included patients aged 12 to 40 years with complete permanent dentition. Axial changes (buccal tilting, oral tilting, mesial tilting, and distal tilting) as well as rotational changes (mesio-buccal rotation and disto-buccal rotation) were investigated. After applying the exclusion criteria, 103 patients remained in the study. Our findings revealed a notable prevalence of positional anomalies, with disto-buccal rotations being most common in upper molars (tooth 1.6–22.3%, tooth 2.6–31.1%) and oral tilting predominating in lower molars (tooth 3.6–6.8%, tooth 4.6–14.6%). Interestingly, neither gender nor malocclusion type significantly influenced the occurrence of tooth position anomalies. Symmetrical patterns in positional changes were observed, with patients having tooth position anomalies at 1.6 significantly more frequently associated with anomalies at 2.6. Similarly, anomalies at 3.6 were significantly more frequently associated with anomalies at 4.6. Additionally, for molars 2.6 and 3.6 on the left side, the differences were statistically significant, with patients having anomalies at 2.6 significantly more frequently associated with anomalies at 3.6. This pattern was not observed for the molars situated on the right side of the dental arches. While this study provides insights into positional anomalies in first permanent molars among the Romanian population, its retrospective design and focus on a specific demographic may limit generalizability. In conclusion, the study underscores the significant prevalence of positional anomalies in first permanent molars among adolescents and adults in Oradea, Romania. Early detection and targeted interventions are crucial to address these anomalies and improve orthodontic outcomes. Comprehensive assessment and treatment planning are essential to achieve optimal dental harmony and function. Further research is needed to elucidate the underlying factors contributing to these positional changes and their long-term impact on oral health and occlusal stability.

## 1. Introduction

Dental anomalies manifest in various forms and can impact the number, size, shape, structure, and position of teeth [1]. Number anomalies include hypodontia or hyperdontia, while size anomalies encompass microdontia and macrodontia [2]. Shape anomalies comprise gemination, fusion, concrescence, additional cusps, dens invaginatus, taurodontism, hypercementosis, dilaceration, and structure anomalies encompass amelogenesis imperfecta, dentinogenesis imperfecta, dentinal dysplasia, and odontodysplasia [2]. Position anomalies include transposition, impaction, and inversion [3], while tooth displacements such as ectopia [3,4], tilting [5], and rotations [6] also fall under the category of positional dental anomalies. These anomalies arise from complex interactions between genetic, epigenetic, and environmental factors during dental development [7]. Anomalies in number, shape, and position typically emerge during the morphodifferentiation stage of tooth development [8].

Factors influencing the position of teeth on dental arches are multifaceted [9]. Vicious oral habits such as finger sucking, nail biting, tongue thrusting, lip biting, and bruxism [10], along with prolonged pacifier use [11] or oral breathing [12], can disrupt the normal position of teeth and alter dental arch shape [9]. However, the risks associated with these habits tend to be more pronounced in genetically susceptible individuals or those with unfavorable growth patterns. Dental trauma and premature extraction of permanent teeth, such as early removal of the first permanent molar, can also induce changes in tooth position [13,14].

The first permanent molars typically erupt around the age of 6 and play a crucial role in establishing correct occlusion [15,16]. However, their position may be affected by ectopic eruption, impaction, or rotations [17,18,19]. Premature extraction of primary second molars, located mesial to the first permanent molar, can lead to the mesial migration or inclination of the first permanent molar, disrupting proper tooth alignment and occlusion [20]. Additionally, the type of Angle malocclusion (Class I, II, or III) can influence the position of permanent first molars [18].

In the Romanian population, malocclusion prevalence is approximately 30% [21], with up to 48% of children experiencing early loss of primary teeth, resulting in positional changes in permanent first molars among certain groups [22]. While ectopic eruption of first permanent molars has been studied in the Romanian population [17], there is a lack of research on rotational and tilting changes in the position of first permanent molars.

Given the critical role of the first permanent molar in establishing proper occlusion and the paucity of data concerning positional changes in this molar type among the Romanian population, this study aimed to identify the most prevalent rotational and axial alterations in first permanent molars among adolescents and adults in Oradea, Romania. Additionally, the study aimed to explore whether there is a gender predisposition for positional changes in first permanent molars and to evaluate symmetry regarding positional alterations among molars situated on the same dental arch or on the hemiarches within the same hemiface.

## 2. Materials and Methods

### 2.1. Ethical Considerations

The study was conducted in accordance with the principles outlined in the Declaration of Helsinki (2008) and its subsequent amendments. Ethical approval was obtained from the Ethics Committee of the Faculty of Medicine and Pharmacy, University of Oradea (IRB No. CEFMF/02 from 30 September 2022).

### 2.2. Sample Selection

This retrospective study involved the examination of digital study models (scans) from a sample of patients in Oradea, Romania. All scans were collected from patients at a Dental Clinic in Oradea. These digital models were created from digital impressions obtained during initial examinations, which were deemed necessary for accurate diagnosis. The scans were captured using the Medit i500 intraoral scanner (Medit Corp., Seoul, Republic of Korea), which was used between 2022 and 2024. The scanning of the patients was conducted by one of three investigators: R.T.M., R.I.J., and A.J.

Informed consent was obtained from all parents or legal guardians for minor patients and from all adult patients, allowing the use of these digital scans for future scientific research. The patients included in the analysis first presented at the dental clinic between 1 September 2022, and 1 May 2024.

Inclusion criteria for the study were as follows: patients aged 12 to 40 years, patients with complete permanent dentition (excluding wisdom teeth), and patients requiring dental treatment. 

Patients were excluded if they had extractions or agenesis of permanent teeth, had previously undergone mobile or fixed orthodontic treatment, had extensive coronal destruction of the first permanent molars, had dental crowns on the first permanent molars, had scans that did not allow proper visualization of the first permanent molars, had local or systemic diseases affecting dento-facial growth and development, or lacked relevant study information.

### 2.3. Examining Changes in Position

**Figure 1:**
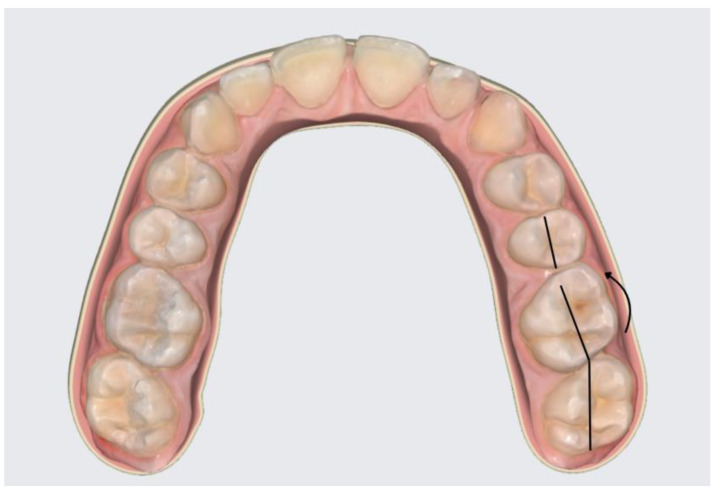
Molar 2.6 with DBR.

**Figure 2:**
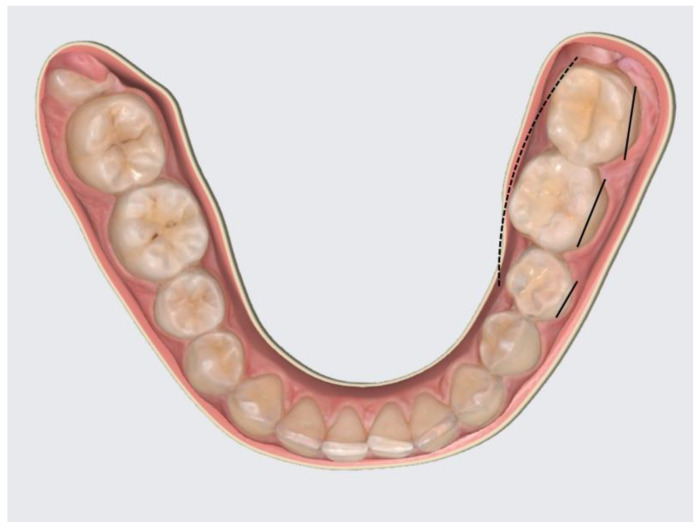
Molar 3.6 with OT.

The study focused on the positional changes of the four first permanent molars: upper right (1.6), upper left (2.6), lower left (3.6), and lower right (4.6). Axial changes, such as buccal tilting (BT—tilting towards the buccal side), oral tilting (OT—tilting towards the oral side), mesial tilting (MT—tilting towards the mesial side), and distal tilting (DT—tilting towards the distal side) of the dental crown, were visually assessed. Rotational changes were categorized as mesio-buccal rotations (MBR—mesial face rotated towards the buccal side) and disto-buccal rotations (DBR—distal face rotated towards the buccal side) (Figure 1 and Figure 2).

The initial diagnosis, according to the Angle classification of malocclusions (Class I, Class II subdivision 1, Class II subdivision 2, Class III), was recorded in the patient files and was used in this study, as well. The diagnosis of Angle malocclusion was determined by either the orthodontists (R.T.M. and A.J.) or the prosthodontist (R.I.J.), and was based on the examination of the patient’s static occlusion, and subsequently confirmed through occlusal scans. The principles established by Edward Angle were employed to categorize the malocclusion as follows:

- Angle Class I Malocclusion: Characterized by a neutral relationship at the level of the first permanent molars, with anomalies present at the level of the incisors.
- Angle Class II/1 Malocclusion: Identified by distal molar occlusion and a positive overjet at the level of the incisors.
- Angle Class II/2 Malocclusion: Marked by distal molar occlusion without an overjet at the level of the incisors.
- Angle Class III Malocclusion: Defined by mesial molar occlusion, with or without a negative overjet at the level of the incisors [23].

The assessment of positional changes on the digital study models was initially performed by one investigator (R.T.M.) and subsequently verified by another investigator (A.E.M.). The inter-rater reliability was 97%, indicating a high level of agreement in diagnosing positional dental anomalies.

### 2.4. Statistical Analysis 

Statistical analysis was conducted using IBM SPSS Statistics 25 (IBM, Chicago, IL, USA) and Microsoft Office Excel/Word 2021 (Microsoft, Redmond, WA, USA). Qualitative variables were expressed as absolute numbers or percentages. Differences between independent qualitative variables were tested using Fisher’s Exact tests. Quantitative variables were expressed as means with standard deviations or medians with interpercentile ranges, with distribution assessed using the Shapiro–Wilk test. Non-parametric quantitative variables were compared between groups using the Mann–Whitney U test. A *p*-value < 0.05 was considered statistically significant.

## 3. Results

### 3.1. Sample Characteristics

Initially, 237 patients were included in the study, but after applying the exclusion criteria, 103 patients remained. Among these, 67 (65%) were female and 36 (35%) were male, with an average age of 18.81 ± 6.25 years. The median age was 16 years, with a minimum age of 12 years and a maximum age of 40 years. Of the patients, 47 (45.6%) were diagnosed with Class I Angle malocclusion, 33 (32%) with Class II malocclusion subdivision 1, 18 (17.5%) with Class II malocclusion subdivision 2, and 5 (4.9%) with Angle Class III malocclusion. Most female patients (35.5%) and male patients (50%) were aged between 12–15 years, while the smallest number of patients were included in the 31–40 years age group (female patients—7.6%, male patients—5.6%) (Table 1). 

### 3.2. Position Anomalies

**Figure 3:**
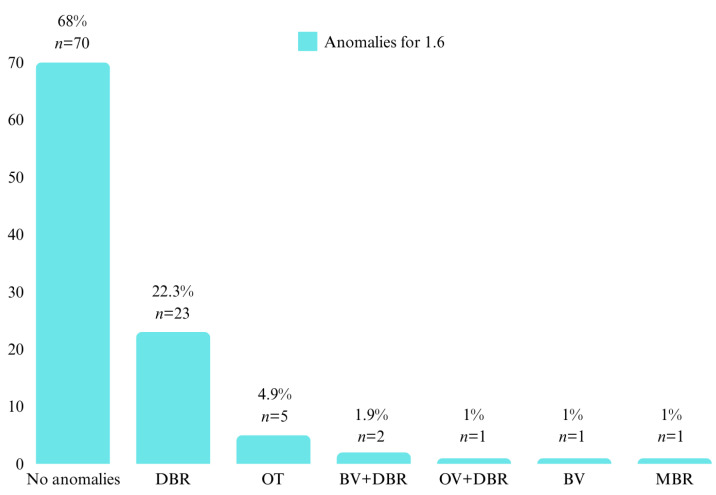
Patient distribution by position anomalies at 1.6.

**Figure 4:**
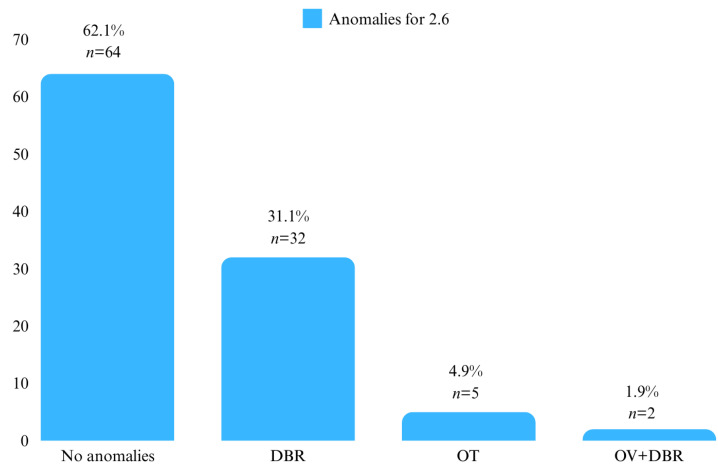
Patient distribution by position anomalies at 2.6.

The positions of the first permanent molars 1.6, 2.6, 3.6, and 4.6 were investigated. For the first permanent molars on the upper arch, it was observed that 32% (*n* = 33) of the patients had position anomalies at molar 1.6, with the most frequent being DBR (*n* = 23, 22.3%). At molar 2.6, 37.9% (*n* = 39) of the patients had position anomalies, with DBR again being the most frequent (*n* = 32, 31.1%) (Figure 3 and Figure 4).

**Figure 5:**
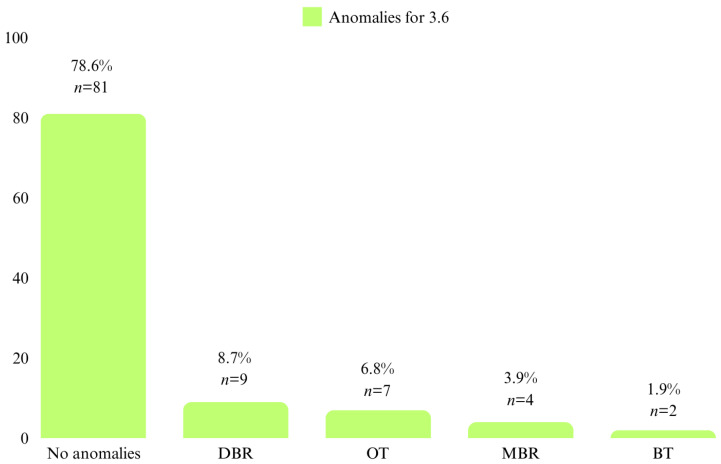
Patient distribution by position anomalies at 3.6.

**Figure 6:**
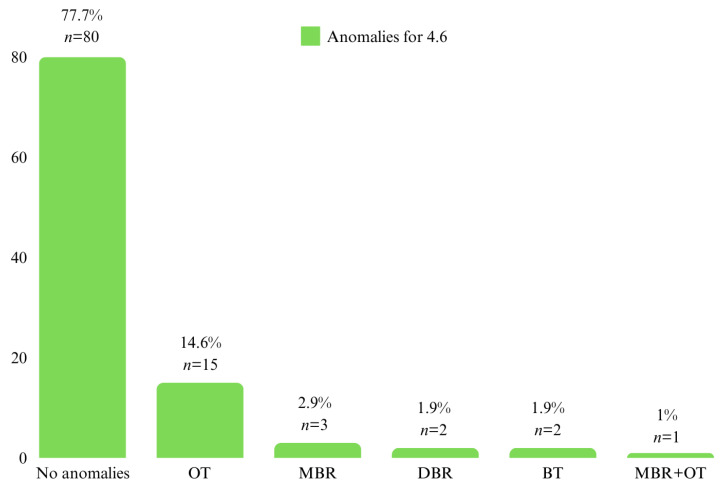
Patient distribution by position anomalies at 4.6.

For the first permanent molars on the lower arch, 21.4% (*n* = 22) of the patients had position anomalies at molar 3.6, with DBR being the most frequent (*n* = 9, 8.7%) followed by OT (*n* = 7, 6.8%). At molar 4.6, 22.3% (*n* = 23) of the patients had position anomalies, with OT being the most frequent (*n* = 15, 14.6%) (Figure 5 and Figure 6).

Possible symmetrical changes in molars on the same arch (upper or lower), as well as molars on the same hemiface (left or right), were also investigated. For molars 1.6 and 2.6 on the upper arch, the differences between the groups were statistically significant according to Fisher’s test (*p* < 0.001), with patients having position anomalies at 1.6 significantly more frequently associated with anomalies at 2.6 (61.5% vs. 14.1%). Similarly, for molars 3.6 and 4.6 on the lower arch, the differences were statistically significant (*p* = 0.001), with anomalies at 3.6 significantly associated with anomalies at 4.6 (47.8% vs. 13.8%) (Table 2).

Regarding molars on the hemiarches of the same hemiface (right or left), it was observed that for molars 1.6 and 4.6 on the right side, the differences were not statistically significant according to Fisher’s test (*p* = 0.079), although there was a tendency towards statistical significance, with higher frequencies of anomalies at 4.6 in patients with anomalies at 1.6 (47.8% vs. 27.5%). However, for molars 2.6 and 3.6 on the left side, the differences were statistically significant (*p* = 0.007), with patients having anomalies at 2.6 significantly more frequently associated with anomalies at 3.6 (63.6% vs. 30.9%) (Table 3).

### 3.3. The Influence of Gender and Type of Malocclusion on Position Anomalies

The data in Table 4 show the distribution of patients according to gender and position anomalies in the four first permanent molars investigated. According to Fisher’s test, the differences between groups were not statistically significant for any of the first permanent molars, indicating that the frequency of positional changes did not significantly differ by patient gender.

Similarly, the distribution of patients according to the type of malocclusion and position anomalies in molars 1.6, 2.6, 3.6, and 4.6 showed no statistically significant differences between groups according to Fisher’s test. Thus, the frequency of position anomalies in these molars was not significantly different based on the type of malocclusion (Table 5).

## 4. Discussion

This study aimed to identify the prevalence and types of positional anomalies in first permanent molars among adolescents and adults in Oradea, Romania. These anomalies have not been extensively studied in the Romanian population, yet they are significant because they can adversely affect occlusal relationships [24] and potentially impact the mechanics of facial growth in children and adolescents [25]. Pathologies associated with positional anomalies of the first permanent molars include posterior crossbite, posterior open-bite caused by primary failure of eruption, and occlusal interferences due to ectopic eruption of the first permanent molars [24,26,27].

The study utilized intraoral digital scans of patients, preferring this method over traditional study models. Intraoral scans offer numerous advantages, including reduced patient discomfort during impressions, precise dental morphology and arch replication, lowered risk of infections, and the ability to easily replicate the scan [28]. These scans are highly accurate, often surpassing the quality of classic dental models, and provide three-dimensional visualization of dental structures and occlusion [29]. The scanner used in this study was the MEDIT i500, which has demonstrated high accuracy [30].

After applying exclusion criteria, the final sample consisted of 103 patients aged between 12 and 40 years. The lower age limit of 12 years was chosen to include patients with complete permanent dentition (excluding the wisdom teeth), ensuring a stable and final position of the permanent first molars, since the eruption of the second permanent molar is typically observed around the age of 12 [31]. The upper age limit of 40 years was selected as it represents the conclusion of the young adulthood stage [32]. The limited number of patients was due to the challenge of identifying individuals with fully dentate arches, excluding the third molars, and possessing good dental health in the first permanent molars. The first permanent molar is typically the first to decay in childhood [33], affecting up to 74% of children and adolescents in certain populations [34]. In Romania, recent studies report a dental caries prevalence of 60.9% in first permanent molars among pediatric populations [35]. Moreover, extraction of first permanent molars is common in late adolescence or adulthood, with rates reaching approximately 40% by the age of 29 [36], and a study by Alshawaf et al. (2023) confirms similar trends in the 31–50 age group, where over 40% of the patients had at least one first permanent molar extracted [37]. Thus, selecting patients who met the inclusion criteria was a critical and challenging process, reflecting the low number of patients with complete dentition.

Regarding positional anomalies, the prevalence at the level of first permanent molars varies from 21.4% at the level of 3.6 to 37.9% at the level of 2.6. Moreover, in the upper arch, the prevalence of positional anomalies in the first permanent molars was higher than in the lower arch. In the upper arch, the most frequent form of positional anomaly was DBR, while in the lower arch, it was OT. Unfortunately, no studies were identified in the specialized literature to allow for comparisons with this study, highlighting an important element of originality in our research.

In this study, neither the type of Angle malocclusion nor the gender of the patients statistically significantly influenced the positional anomalies identified in the first permanent molars. This suggests that positional anomalies might be influenced more by local dental arch factors and early childhood habits rather than broader demographic factors [38]. The study by Viganó et al. (2016) was the only one identified that examined the rotations of first permanent molars in Class I, II, or III Angle malocclusions [19]. While DBR was the most frequent anomaly identified by Viganó et al., defined as mesio-palatal rotation [19], our study also found DBR to be the most common positional anomaly in upper first permanent molars.

The symmetry of the positional anomalies identified in this study was also noteworthy. Molars in the upper arch (1.6 and 2.6) and those in the lower arch (3.6 and 4.6) were symmetrically affected in a statistically significant manner, as were the molars on the hemiarches on the left side of the face (2.6 and 3.6). No comparative studies were found in the specialized literature for these results, further underscoring the originality of this study.

This study introduces several novel insights into dental positional anomalies. Notably, it is among the first to provide detailed prevalence data on rotational and tilting anomalies in the first permanent molars of a Romanian population, highlighting the significant occurrence of DBR in upper molars and OT in lower molars. Additionally, the use of digital models for precise measurement of these anomalies represents a methodological advancement, ensuring high accuracy and reliability. The findings also contribute to the limited body of knowledge on the lack of significant gender and malocclusion type differences in these positional anomalies, suggesting that other factors may play a more critical role.

### Limitations and Clinical Implications

This study has several limitations that must be acknowledged. The retrospective design limits the ability to infer causality. Focusing solely on a specific population in Oradea may not fully represent the results’ applicability to other regions or ethnic groups. This regional focus could potentially limit the broader relevance of our findings in different demographic settings, where genetic, environmental, and socio-economic factors might diverge significantly.

Potential selection biases and the absence of longitudinal data hinder a comprehensive understanding of the progression and long-term impacts of tooth position anomalies. These factors may reduce the accuracy of predicting long-term outcomes based on our findings. Addressing these limitations in future research should include more diverse populations and employ longitudinal study designs to observe the progression of position anomalies over time.

The wide age range of participants, while necessary to maintain a sufficient sample size, introduces another potential source of bias. The variability in age could affect the developmental stage of dental health and the prevalence of anomalies, possibly skewing the results. Furthermore, the predominance of female patients, potentially attributed to better oral hygiene practices and a higher propensity for dental visits [39], might introduce gender bias in the findings. These demographic nuances are crucial for clinicians when considering the broader applicability of the study results.

Clinically, these limitations suggest cautious interpretation when applying these findings to patient care, especially in diverse settings. Practitioners should consider these factors when diagnosing and planning treatments based on this study’s data, potentially limiting the direct application of our conclusions in universal clinical contexts.

Future studies could enhance the practical relevance of this research by exploring the specific clinical impacts of identified tooth position anomalies, thereby aiding in the development of targeted intervention strategies. By directly linking observed position anomalies with specific clinical outcomes, subsequent research can offer more definitive guidance for dental practitioners, ultimately improving patient care and treatment outcomes.

## 5. Conclusions

In conclusion, this study reveals a significant prevalence of DBR in upper first permanent molars and OT in lower molars among the population in Oradea, Romania. These findings underscore the importance of early detection and targeted interventions to improve orthodontic outcomes. The use of digital models for precise measurement is a methodological strength, ensuring high accuracy and reliability. The study’s observation of symmetrical positional anomalies in both upper and lower molars, along with left hemiarch molars, underscores its originality and highlights a gap in existing comparative literature. The findings of this study, while insightful, must be viewed in light of its limitations such as its retrospective design and focus on a specific region, which may affect the broader applicability of the results. Additionally, the wide age range and higher number of female participants could skew the observed prevalence and characteristics of tooth position anomalies. Clinically, this research can enhance orthodontic diagnostics and treatment planning. The identification of patterns in DBR and OT can guide clinicians in similar settings, and the use of digital models promotes more accurate orthodontic interventions.

## Figures and Tables

**Table 1:** Patient distribution according to gender and age group.

**Female Patients**		
**Age Group**	**No.**	**%**
12–15 years	24	35.8%
16–20 years	20	29.8%
21–30 years	18	26.8%
31–40 years	5	7.6%
**Male Patients**		
12–15 years	18	50%
16–20 years	9	25%
21–30 years	7	19.4%
31–40 years	2	5.6%

**Table 2:** Patient distribution by position anomalies for the upper arch and lower arch first permanent molars.

**1.6 and 2.6**					
**PA**	**2.6—Without PA**		**2.6—With PA**		*p* *
	**No.**	**%**	**No.**	**%**	
1.6—Without PA	55	85.9%	15	38.5%	<0.001
1.6—With PA	9	14.1%	24	61.5%	
**3.6 and 4.6**					
**PA**	**4.6—Without PA**		**4.6—With PA**		
3.6—Without PA	69	86.3%	12	52.2%	0.001
3.6—With PA	11	13.7%	11	47.8%	

PA—Position Anomalies, * Fisher’s Exact Test.

**Table 3:** Patient distribution by position anomalies for the right and left first permanent molars.

**1.6 and 4.6**					
**PA**	**4.6—Without PA**		**4.6—With PA**		*p* *
	**No.**	**%**	**No.**	**%**	
1.6—Without PA	58	72.5%	12	52.2%	0.079
1.6—With PA	22	27.5%	11	47.8%	
**3.6 and 4.6**					
**PA**	**3.6—Without PA**		**3.6—With PA**		
2.6—Without PA	56	69.1%	8	36.4%	0.007
2.6—With PA	25	30.9%	14	63.6%	

PA—Position Anomalies, * Fisher’s Exact Test.

**Table 4:** Patient distribution according to gender and position anomalies.

	Gender/ PA	Female		Male		*p* *
		No.	%	No.	%	
1.6	No	45	67.2%	25	69.4%	1.000
	Yes	22	32.8%	11	30.6%	
2.6	No	40	59.7%	24	66.7%	0.529
	Yes	27	40.3%	12	33.3%	
3.6	No	51	76.1%	30	83.3%	0.458
	Yes	16	23.9%	6	16.7%	
4.6	No	52	77.6%	28	77.8%	1.000
	Yes	15	22.4%	8	22.2%	

PA—Position Anomalies, * Fisher’s Exact Test.

**Table 5:** Patient distribution according to malocclusion and position anomalies.

	Malocclusion/ PA	Class I		Class II/1		Class II/2		Class III		*p* *
		No.	%	No.	%	No.	%	No.	%	
1.6	No	35	74.5%	19	57.6%	12	66.7%	4	80%	0.430
	Yes	12	25.5%	14	42.4%	6	33.3%	1	20%	
2.6	No	33	70.2%	16	48.5%	12	66.7%	3	60%	0.247
	Yes	14	29.8%	17	51.5%	6	33.3%	2	40%	
3.6	No	38	80.9%	24	72.7%	14	77.8%	5	100%	0.620
	Yes	9	19.1%	9	27.3%	4	22.2%	0	0%	
4.6	No	38	80.9%	26	78.8%	13	72.2%	3	60%	0.597
	Yes	9	19.1%	7	21.2%	5	27.8%	2	40%	

PA—Position Anomalies, * Fisher’s Exact Test.

## Data Availability

The data presented in this study are available on request from the corresponding authors. The data are not publicly available due to privacy reasons.
